# Undifferentiated Castration‐Resistant Prostate Cancer Successfully Treated With Carboplatin and Paclitaxel

**DOI:** 10.1155/criu/1722086

**Published:** 2026-02-27

**Authors:** Masayuki Sano, Masaki Shimbo, Shin Ohgita, Naoki Kanomata, Kazunori Hattori, Fumiyasu Endo

**Affiliations:** ^1^ Department of Urology, St. Luke′s International University, Tokyo, Japan; ^2^ Department of Clinical Oncology, St. Luke′s International University, Tokyo, Japan; ^3^ Department of Pathology, St. Luke′s International University, Tokyo, Japan

## Abstract

**Introduction:**

Castration‐resistant prostate cancer (CRPC) is a heterogeneous and aggressive disease with limited treatment options, particularly in poorly differentiated cases that lack androgen receptor expression and progress rapidly even under conventional therapies. Here, we present a rare case of poorly differentiated CRPC that exhibited a remarkable response to carboplatin and paclitaxel therapy.

**Case Presentation:**

A 53‐year‐old man with metastatic, castration‐sensitive prostate cancer (cT3bN1M1b, PSA 9.39 ng/mL, Gleason 4 + 5) underwent androgen deprivation therapy using bicalutamide and leuprorelin. Nine months later, he developed CRPC, despite undetectable PSA levels. A supraclavicular lymph node biopsy revealed a poorly differentiated carcinoma. Immunohistochemistry was negative for PSA and NKX3.1 and positive for CK5/6. These results excluded neuroendocrine carcinoma and supported a diagnosis of undifferentiated carcinoma. Systemic chemotherapy with carboplatin (area under the curve, five) and paclitaxel (200 mg/m^2^) was initiated. Partial remission was achieved at 2 months, and complete remission was confirmed at 18 months. After 73 cycles, chemotherapy was discontinued, and the patient has remained in remission during follow‐up.

**Conclusion:**

This case highlights the potential efficacy of carboplatin and paclitaxel in treating poorly differentiated CRPC, suggesting the need for further investigation into platinum‐based regimens for aggressive prostate cancer variants.

## 1. Introduction

Castration‐resistant prostate cancer (CRPC) is an advanced, aggressive form of prostate cancer that progresses despite androgen deprivation therapy [1, 2]. CRPC is characterized by continued androgen receptor (AR) signaling through various mechanisms, including autocrine androgen production and AR activation despite low testosterone levels [3]. This disease presents a spectrum from rising prostate‐specific antigen (PSA) levels without metastases to symptomatic metastatic disease, primarily affecting bones and lymph nodes [2]. Recent advancements in understanding CRPC biology have led to the development of new targeted therapies, including AR pathway inhibitors, immunotherapy, and chemotherapy [4, 5]. Despite these improvements, CRPC remains incurable, necessitating ongoing research into novel therapeutic targets and biomarkers to improve patient outcomes [5, 6].

Poorly differentiated CRPC presents significant treatment challenges owing to its rapid progression and the low/absent expression of ARs [7]. It is characterized by rapid progression, visceral metastases, and hormone refractoriness, often with low PSA levels [7]. Aggressive variant prostate cancer and neuroendocrine prostate cancer are AR‐independent subtypes that often emerge after androgen deprivation therapy in patients with CRPC [8, 9]. These aggressive variants are characterized by rapid disease progression, hormone refractoriness, and poor prognoses [10]. Treatment options for CRPC include androgen deprivation therapy, chemotherapy, immunotherapy, radiation therapy, and poly ADP‐ribose polymerase inhibitors [11]. Despite these therapeutic options, the prognosis for poorly differentiated CRPC remains poor, highlighting the need for ongoing research and the identification of predictive biomarkers [12]. Carboplatin and paclitaxel combination therapy has shown efficacy in treating CRPC, particularly in heavily pretreated patients. Studies have reported PSA level response rates of 42%–69% and median progression‐free survival of 12–16 weeks [13, 14]. However, most cases did not achieve a complete remission because of the inherent challenges associated with disease progression and treatment resistance.

Herein, we report a case of poorly differentiated metastatic CRPC that exhibited a remarkable response to carboplatin and paclitaxel therapies.

## 2. Case Presentation

The patient was a 53‐year‐old man who was referred to our institution after undergoing a prostate biopsy at another hospital, which revealed prostate adenocarcinoma with a Gleason score of 4 + 5. Staging studies showed a locally advanced disease, with seminal vesicle invasion, as well as pelvic lymph node and bone metastases (Figure 1). He was diagnosed with metastatic prostate cancer (initial PSA: 9.39 ng/mL, cT3bN1M1b), and received hormone therapy with bicalutamide tablets 80 mg once a day and leuprorelin 22.5 mg subcutaneous injection once every 6 months. His PSA levels rapidly decreased to undetectable levels. Six months posttreatment initiation, metastasis to the ischiopubic ramus was identified, requiring palliative radiotherapy (Figure 2). Three months later, multiple lymph node metastases and metastases to the right iliopsoas muscle were detected, necessitating an additional course of palliative radiotherapy (Figure 3).

Figure 1Magnetic resonance imaging showing seminal vesicle invasion (a) and initial metastases to the pelvic lymph nodes (b).

**Figure:**
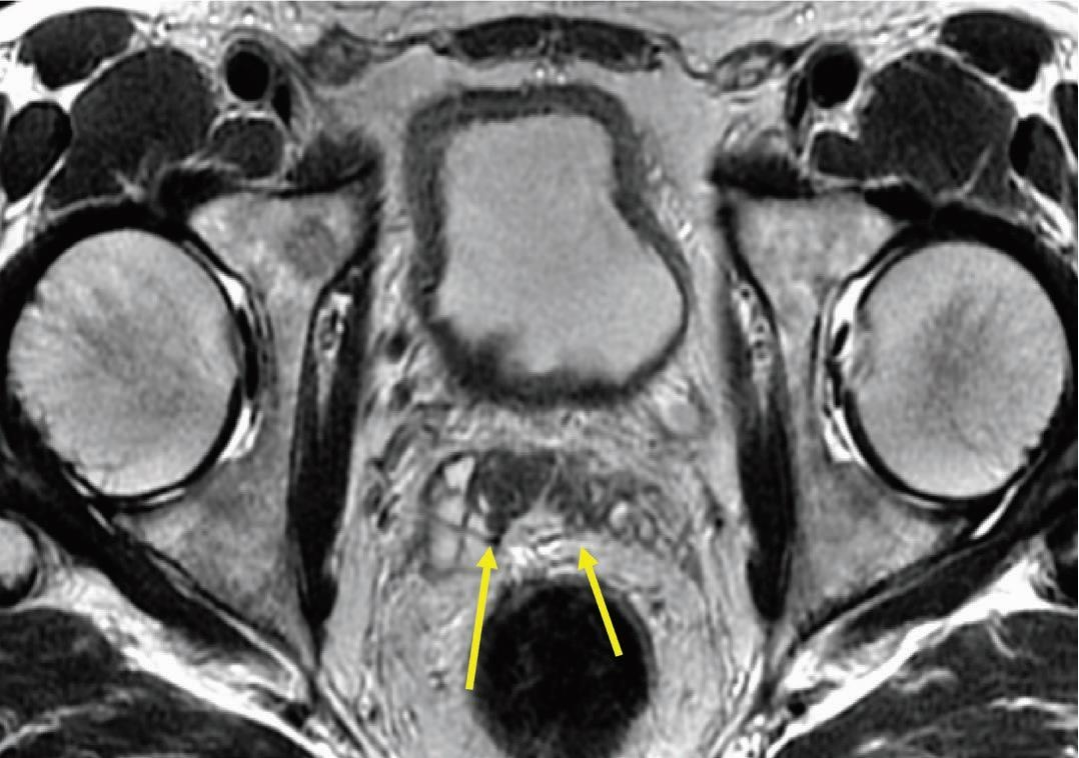
(a)

**Figure:**
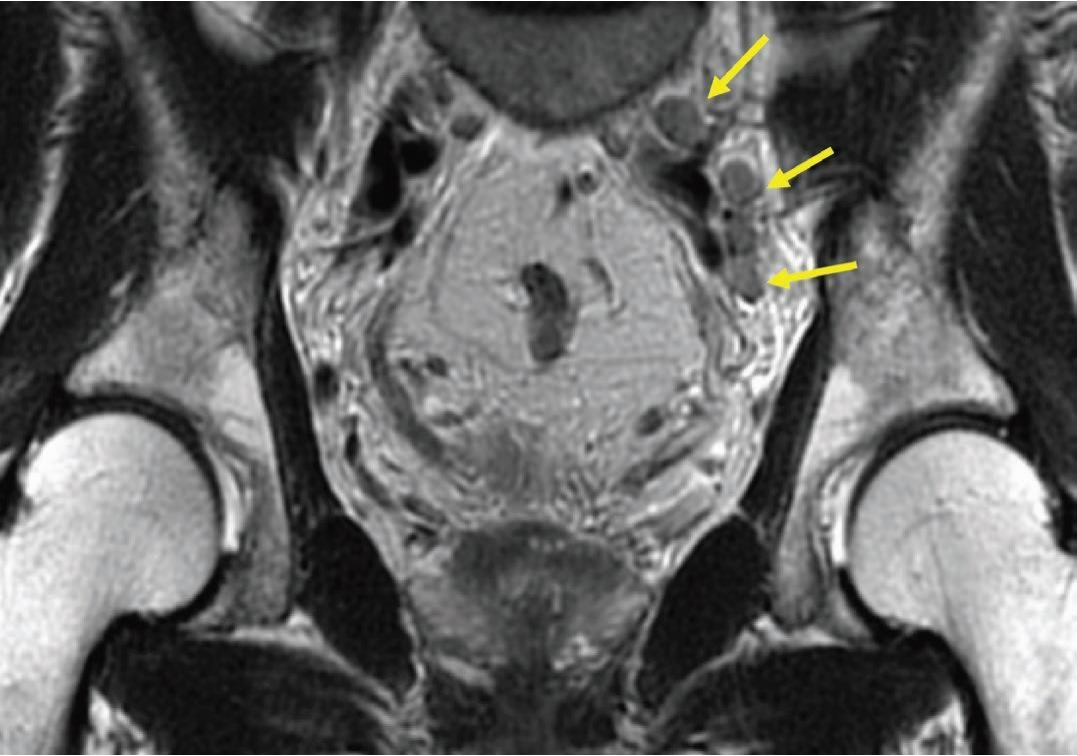
(b)

Figure 2Magnetic resonance imaging (a) and bone scan (b) indicate the progression of bone metastases before initiating carboplatin and paclitaxel.

**Figure:**
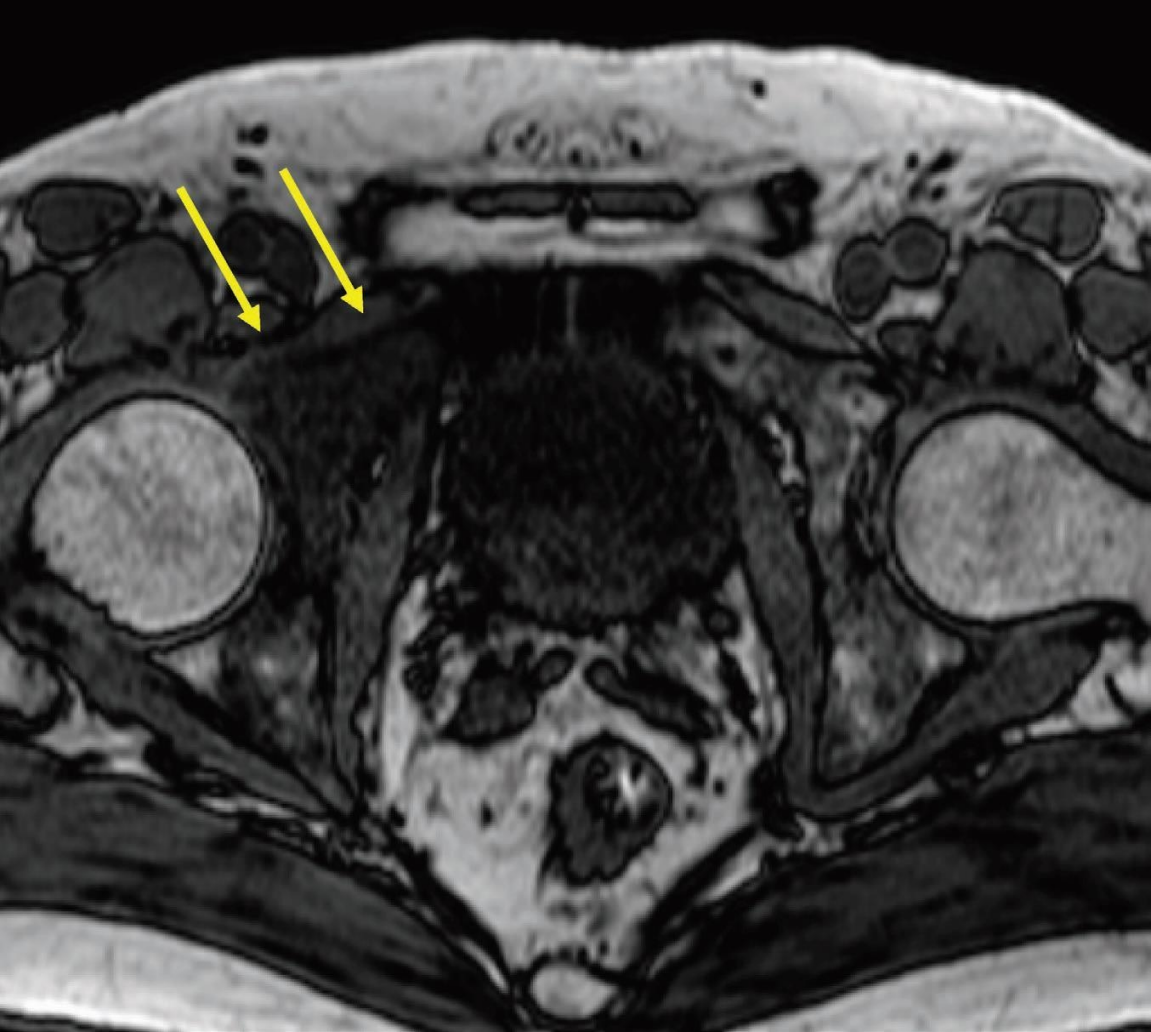
(a)

**Figure:**
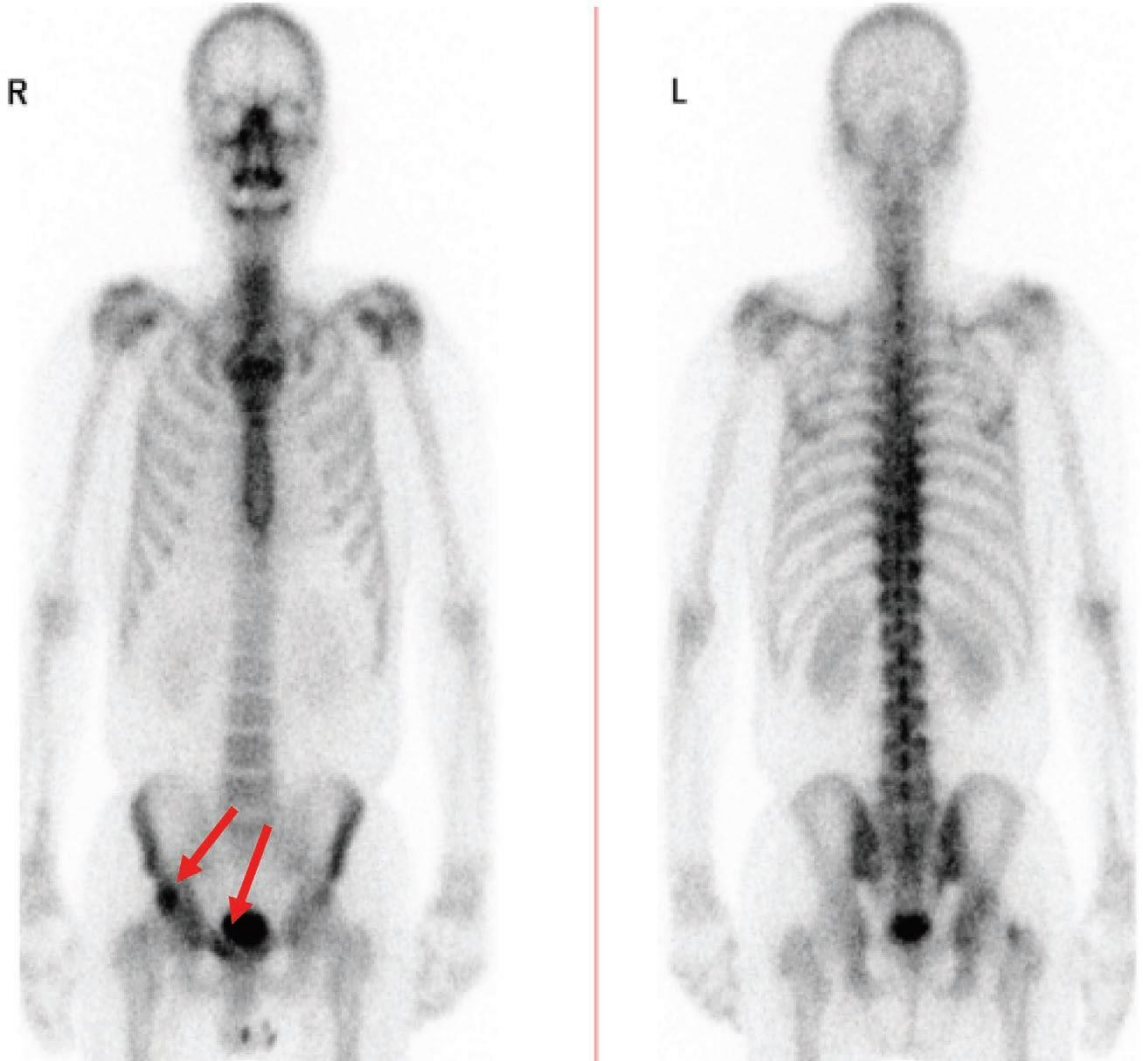
(b)

Figure 3Computed tomography images demonstrating (a) left subclavian lymph node metastasis, (b) left para‐aortic lymph node metastasis, and (c) right iliopsoas muscle metastasis.

**Figure:**
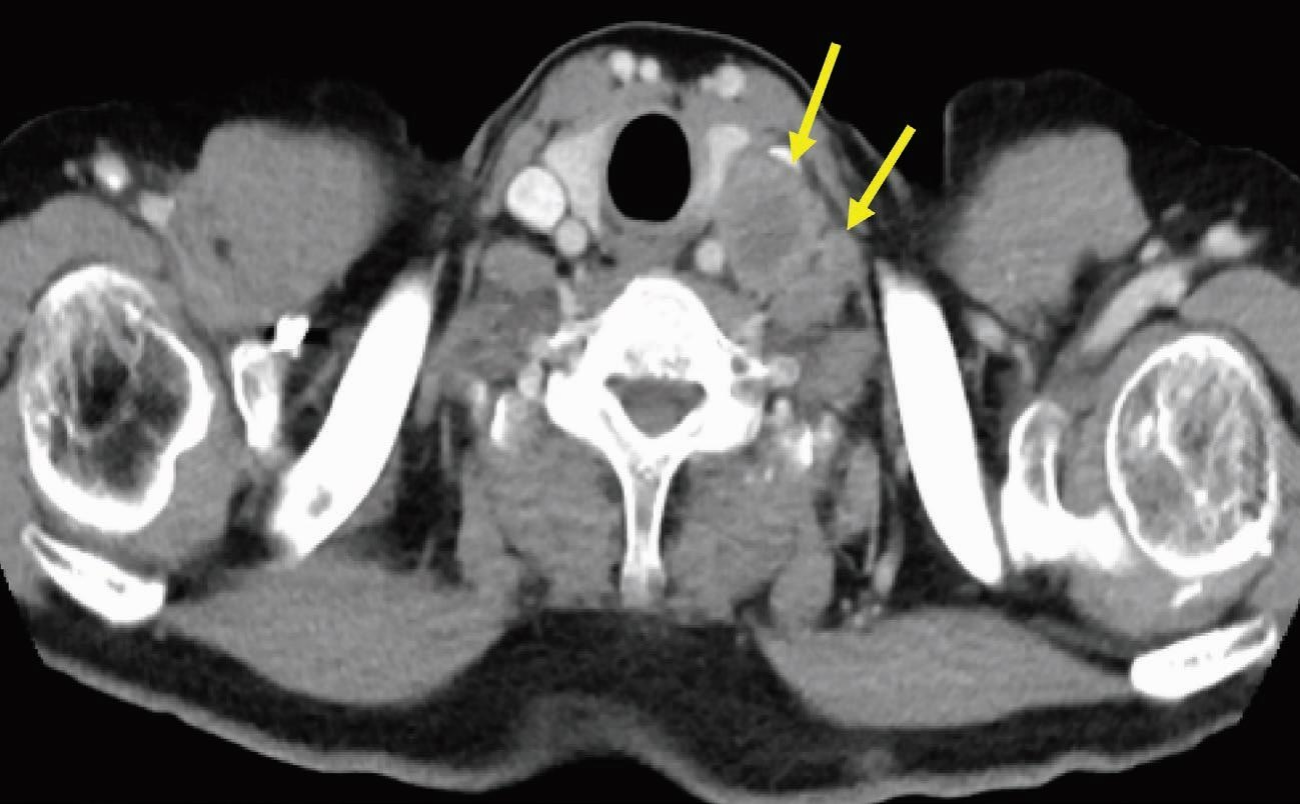
(a)

**Figure:**
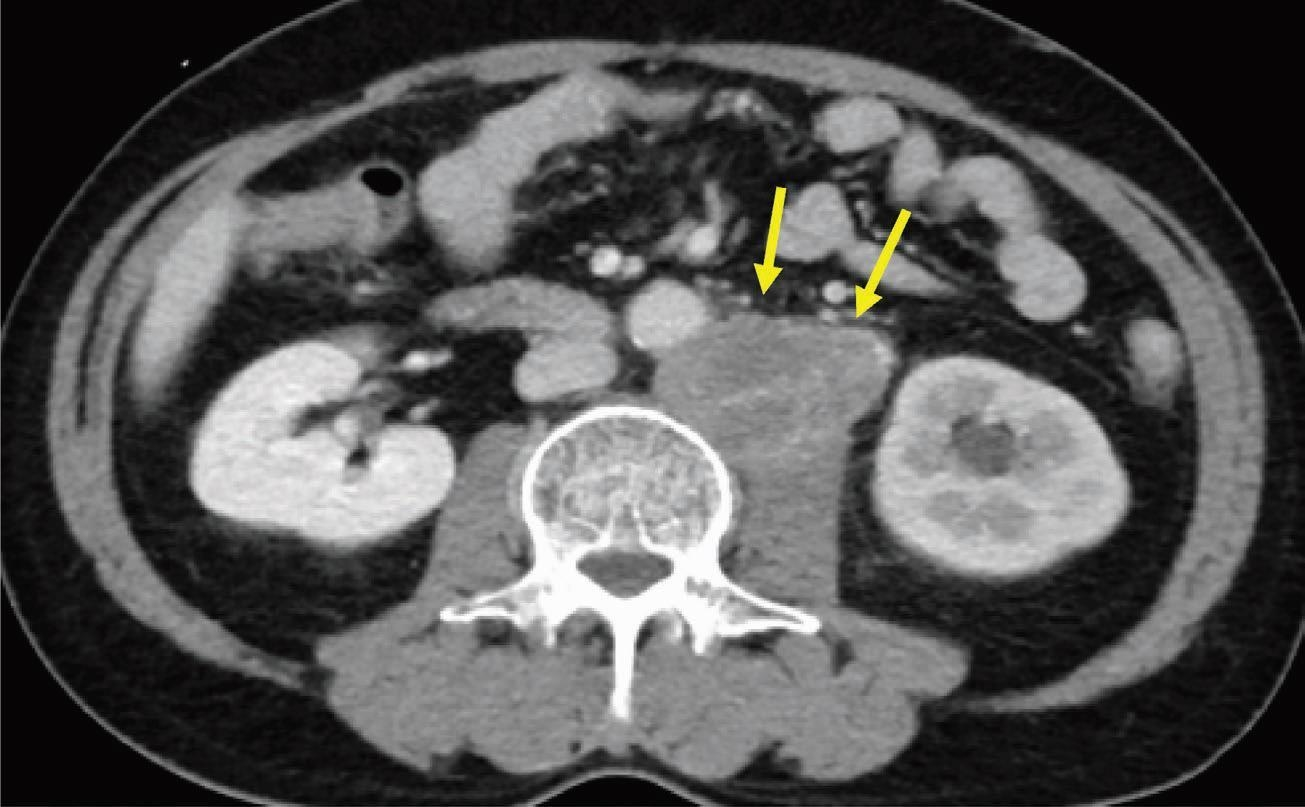
(b)

**Figure:**
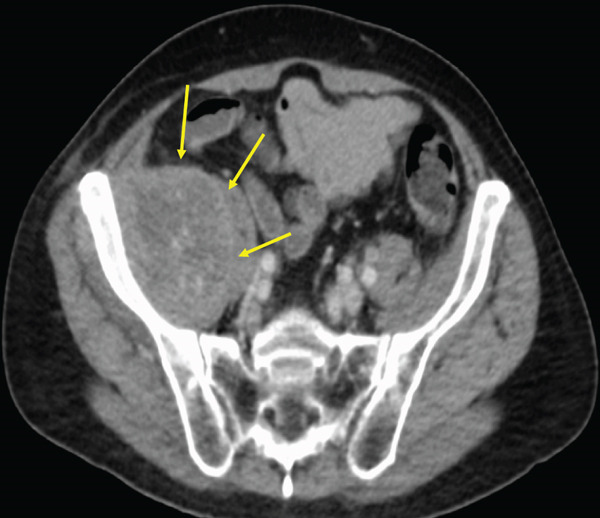
(c)

Despite the clinical suspicion of progressive prostate cancer, PSA levels remained below the detectable thresholds. Supraclavicular lymph node biopsy revealed an undifferentiated adenocarcinoma. Immunohistochemical staining was negative for PSA and NKX3.1, whereas CK5/6 was positive, indicating a significant deviation from prostate cancer (Figure 4). The neuron‐specific enolase level was elevated at 282 ng/mL, whereas Chromogranin A and synaptophysin were negative, excluding neuroendocrine differentiation.

Figure 4The undifferentiated adenocarcinoma, as identified on a supraclavicular lymph node biopsy. (a) Hematoxylin and eosin (HE) staining showing multiple solid clusters of atypical cells within the soft tissue, accompanied by lymphocytes. Some clusters exhibited central necrosis. (b, c, and d) Immunohistochemical staining showing that PSA (b) and NKX3.1 (c) were negative, whereas CK5/6 (d) was positive, indicating a poorly differentiated carcinoma.

**Figure:**
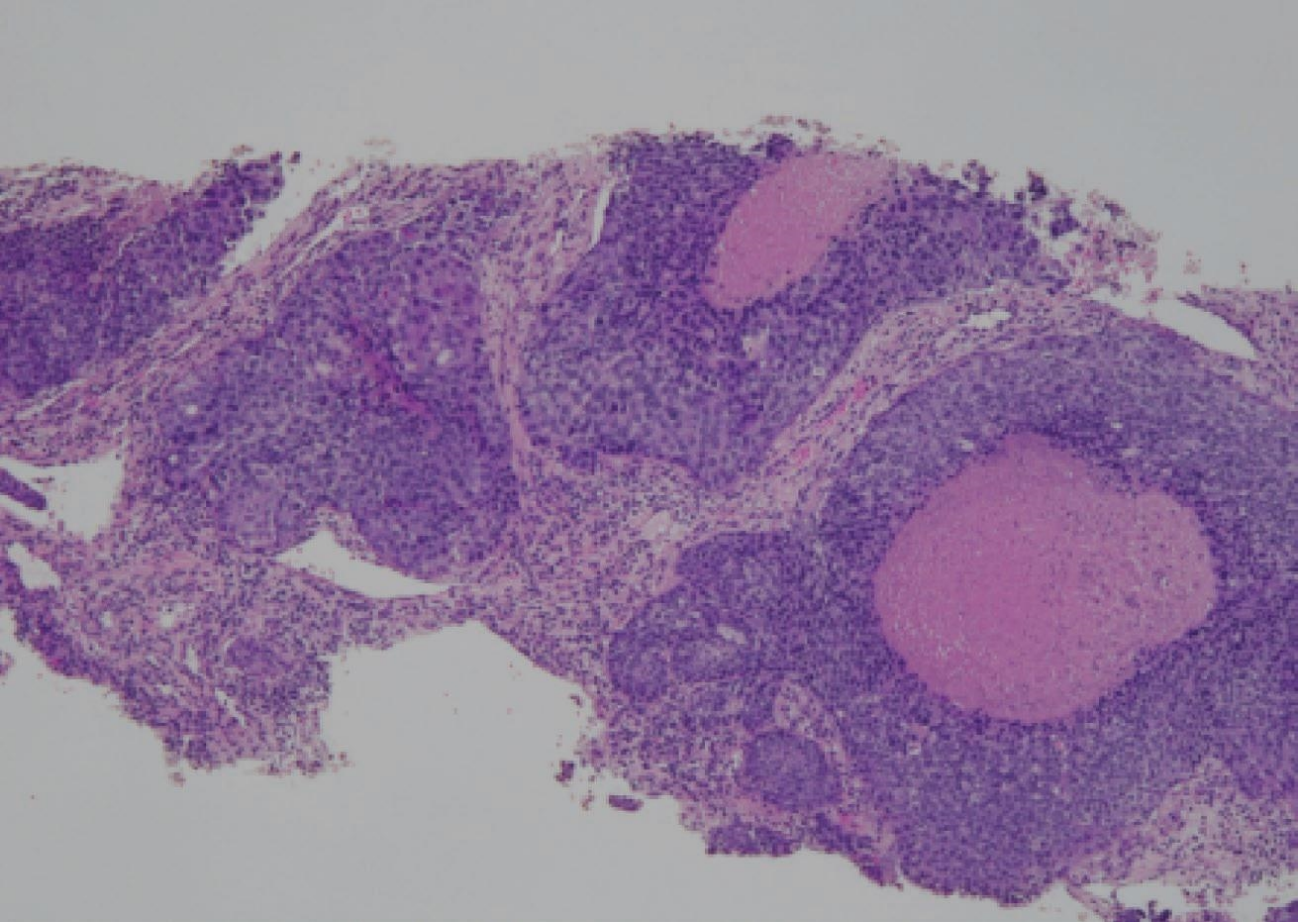
(a)

**Figure:**
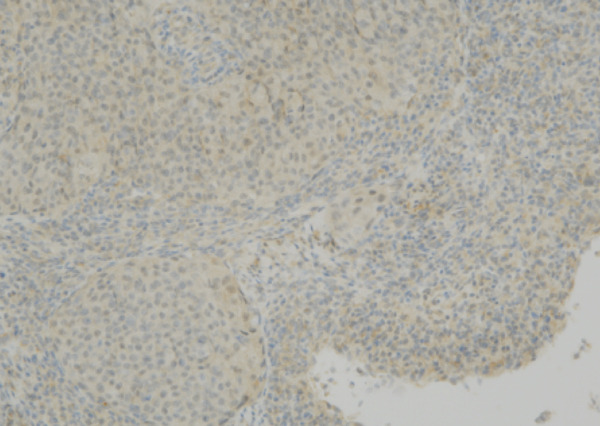
(b)

**Figure:**
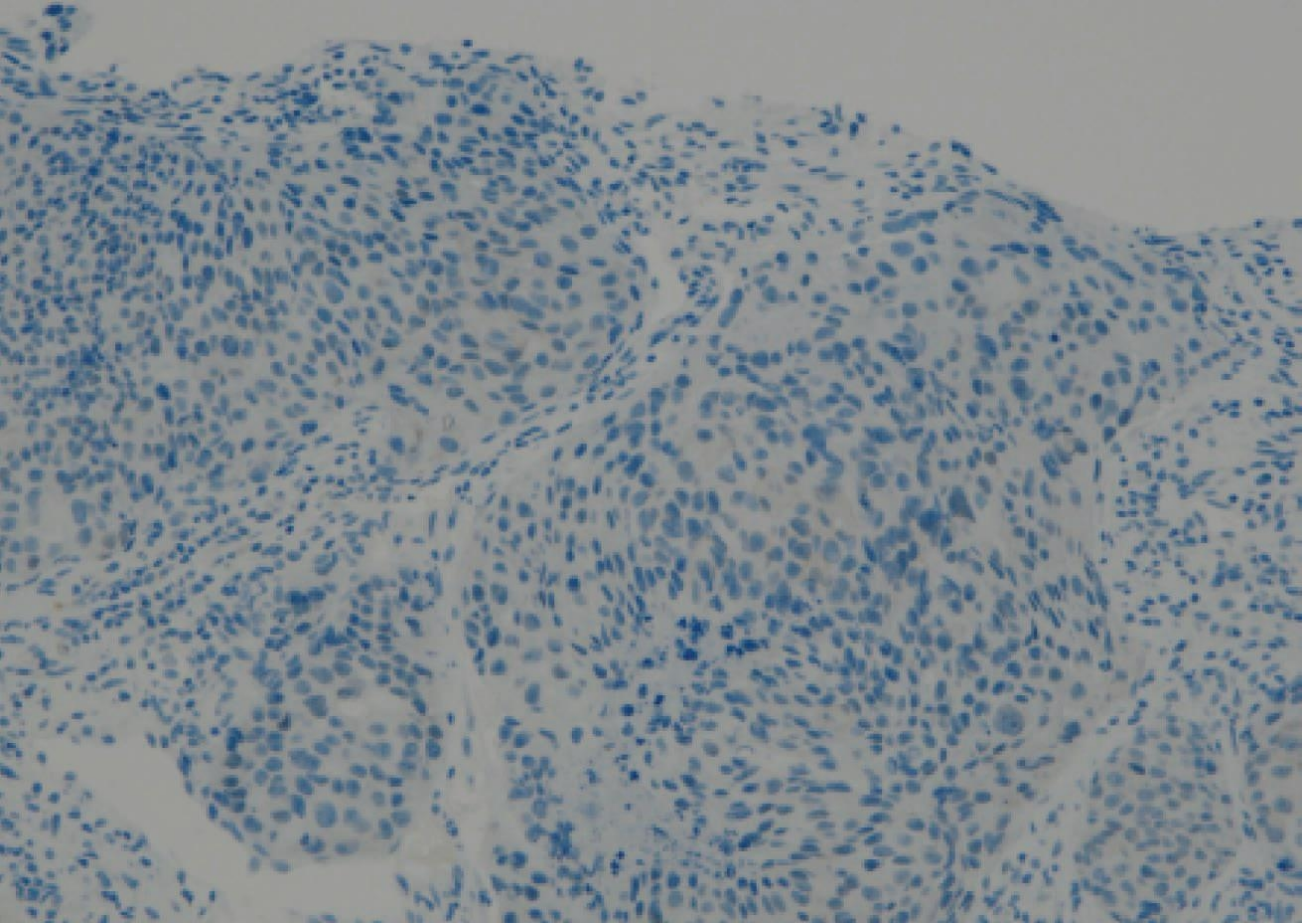
(c)

**Figure:**
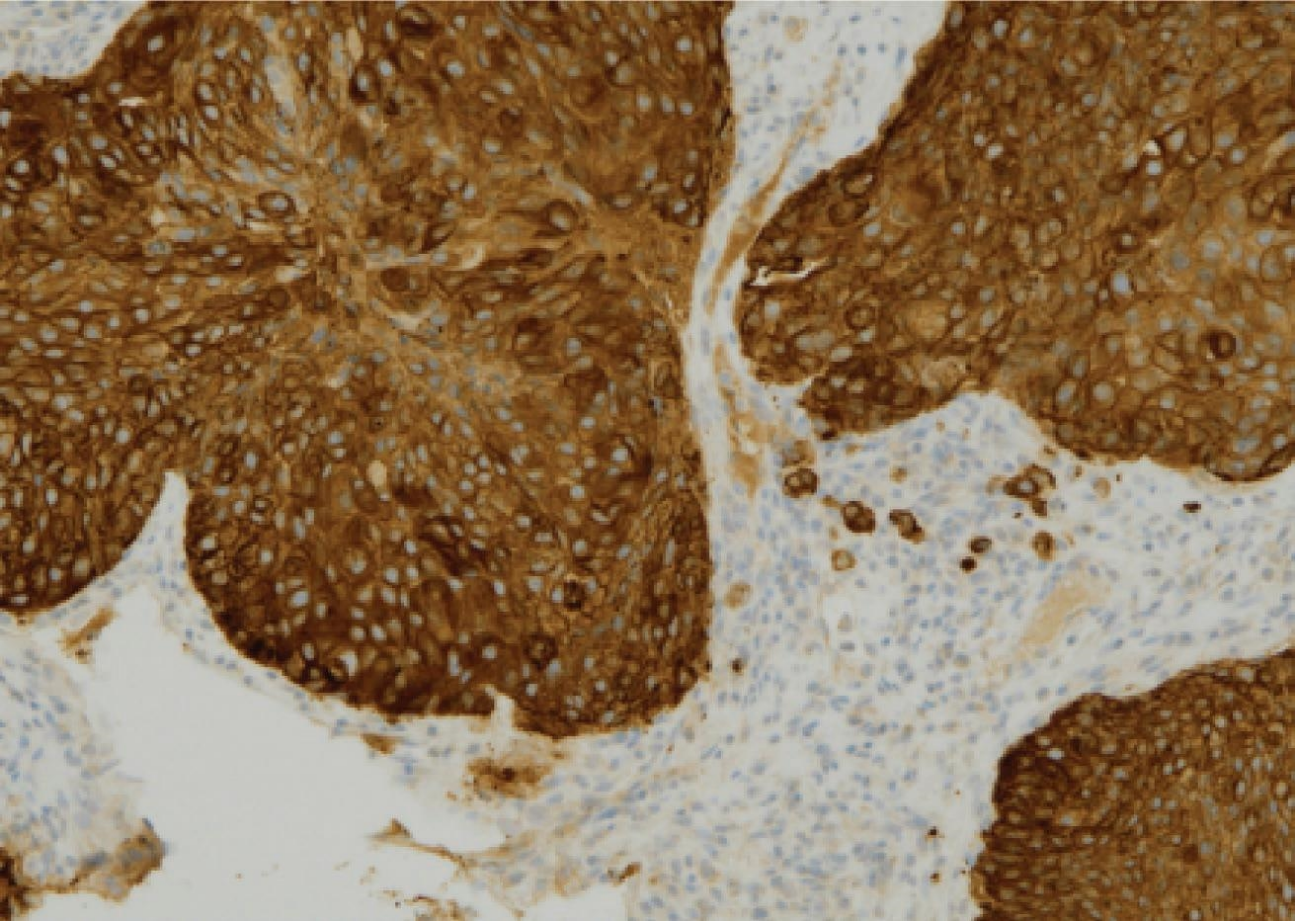
(d)

Given the rapid disease progression and atypical aggressiveness following the initiation of androgen deprivation therapy with bicalutamide and leuprorelin, standard sequential therapies, such as androgen receptor signaling inhibitors (ARSIs) or docetaxel, with or without radiotherapy, were considered, but ultimately not pursued. Supraclavicular lymph node biopsy was promptly performed to clarify the disease phenotype, revealing a poorly differentiated carcinoma with histological features suggestive of dedifferentiation. Based on these findings, systemic platinum‐based chemotherapy with carboplatin and paclitaxel was initiated as treatment for carcinoma of unknown primary origin, rather than proceeding with second‐line hormonal agents such as ARSIs.

The regimen included carboplatin (AUC 5 mg/mL/min) and paclitaxel (200 mg/m^2^) administered every 3 weeks. Partial remission was observed 2 months following the commencement of chemotherapy. Complete remission was confirmed at the 18‐month follow‐up visit, as evidenced by a computed tomography scan demonstrating the absence of detectable disease (Figure 5). Subsequently, 73 courses of chemotherapy were administered, and the treatment was discontinued. The patient has remained in remission without any evidence of recurrence.

Figure 5Computed tomography scan demonstrating complete remission after carboplatin and paclitaxel treatment.

**Figure:**
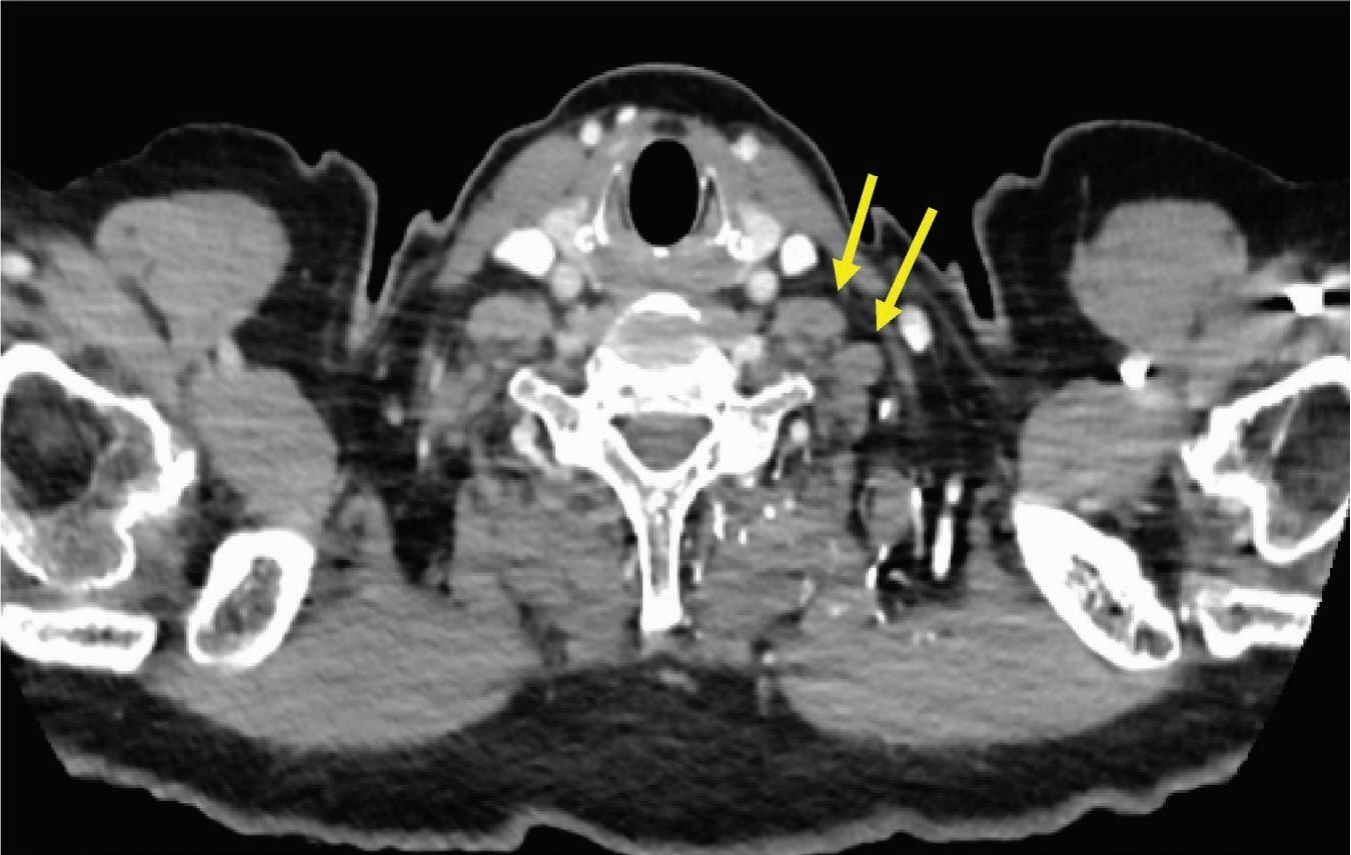
(a)

**Figure:**
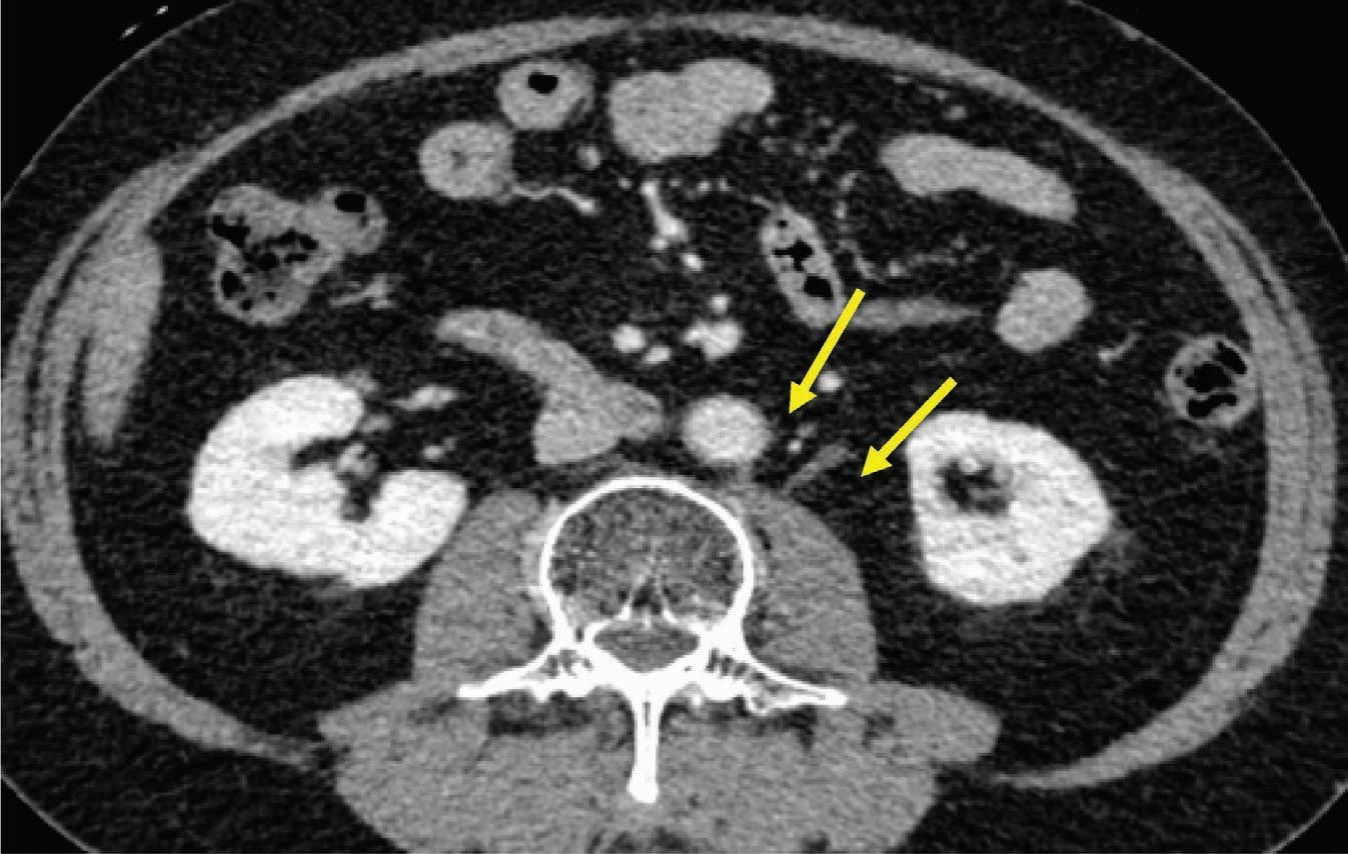
(b)

**Figure:**
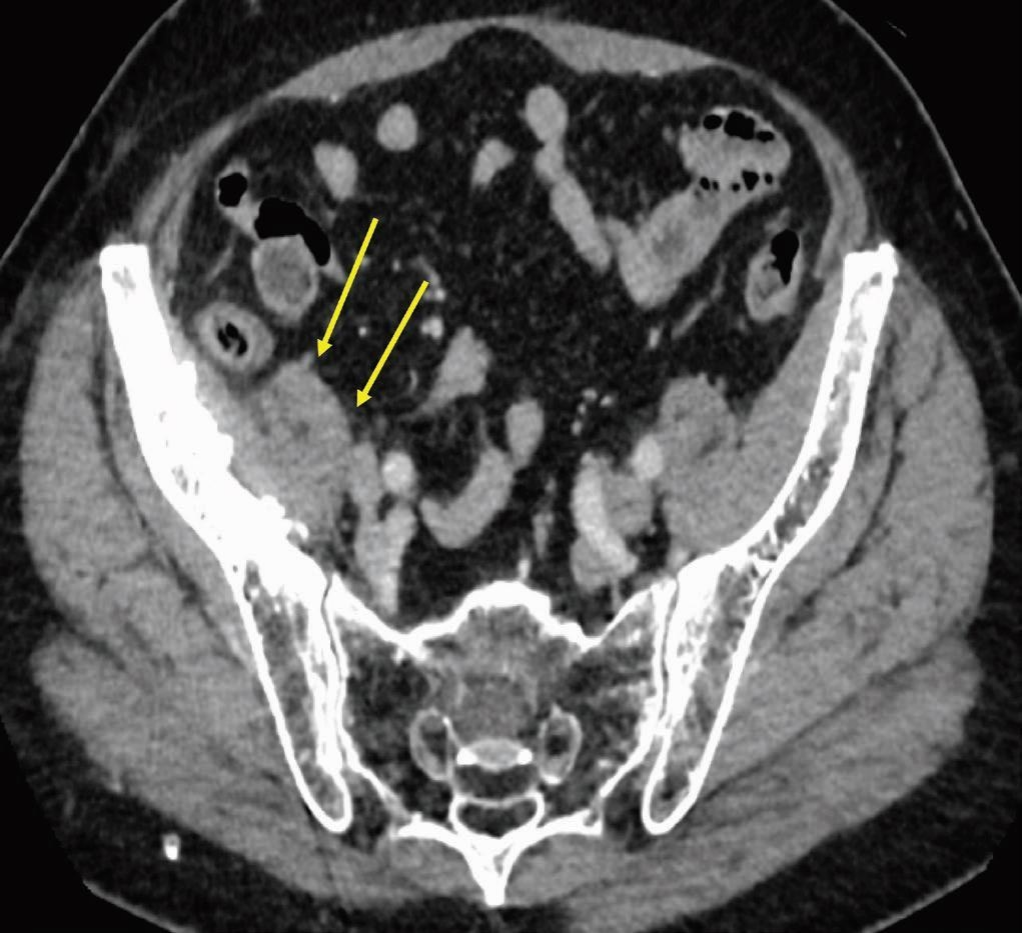
(c)

Treatment‐related adverse events, assessed according to the Common Terminology Criteria for Adverse Events (CTCAE), Version 5.0, were carefully monitored. The patient experienced Grade 1 peripheral neuropathy, mild peripheral edema, and Grade 1 hematological toxicity, all of which were manageable with supportive care and did not necessitate any dose reduction or treatment discontinuation. No Grade 3 or higher adverse events were observed, and the patient′s quality of life was maintained throughout chemotherapy.

## 3. Discussion

This case underscores the importance of exploring alternative therapeutic regimens for patients with poorly differentiated CRPC presenting with atypical features. Despite the supraclavicular lymph node biopsy exhibiting an absence of definitive characteristics associated with conventional prostate adenocarcinoma, the initial prostate needle biopsy revealed a Gleason Grade 5 tumor in a patient with low serum PSA levels. This pattern initially suggested the potential for neuroendocrine differentiation. Although the presence of a concurrent cancer of unknown primary could not be entirely ruled out, the clinical and histopathological findings supported the interpretation of a dedifferentiated or neuroendocrine‐transformed prostate cancer rather than a second malignancy. The combination of carboplatin and paclitaxel demonstrated significant efficacy, supporting the potential role of this regimen in managing similar cases.

Several studies have reported the efficacy and tolerability of this combination therapy in patients with metastatic CRPC [13, 14]. For example, one case study documented durable control of metastatic CRPC with the early introduction of carboplatin and paclitaxel [15]. Furthermore, this combination has also proven effective and well‐tolerated in patients with advanced urothelial carcinoma [16, 17].

In the present case, a 53‐year‐old man with undifferentiated, early CRPC exhibited a remarkable response to carboplatin and paclitaxel therapy. This observation adds to the growing evidence supporting the effectiveness of this chemotherapeutic regimen for the treatment of aggressive prostate cancer. However, due to the patient′s deteriorating condition, rebiopsy of the prostate was not a viable option. Consequently, a direct histopathological comparison between the primary tumor and the metastatic lymph node lesion could not be performed. This limitation precludes any definitive conclusions regarding the origin of the supraclavicular lesion. In the future, cases exhibiting similar presentations may benefit from multisite pathological evaluation to clarify the origin and biology of the disease.

The therapeutic efficacy observed in the present case can likely be attributed to several underlying mechanisms previously described in the literature. One potential explanation involves the DNA damage response pathway. Carboplatin, a platinum‐based chemotherapeutic agent, is particularly effective against tumors with defects in DNA repair mechanisms, such as those involving the *BRCA2* gene. Studies have further shown that mutations in the *BRCA2* gene can result in increased sensitivity to platinum‐based therapies, such as carboplatin, owing to the impaired ability of cancer cells to repair DNA crosslinks, ultimately leading to cell death [18].

Additionally, paclitaxel, a microtubule‐stabilizing agent, inhibits cell division, which is particularly advantageous in combating the rapidly proliferating cells characteristic of poorly differentiated cancers. The synergistic effect of carboplatin and paclitaxel enhances the cytotoxic effects on cancer cells, as observed in prior studies evaluating this combination therapy in CRPC [14].

Aggressive variants of CRPC, which are characterized by high‐grade features and treatment resistance and often exhibit neuroendocrine characteristics or anaplastic morphology, have been found to respond more favorably to platinum‐based therapies. This responsiveness may stem from the unique biology of these tumor types, including their rapid growth rates and distinct molecular alterations, rendering them more susceptible to DNA‐damaging agents [1].

The treatment strategy we used differed from the standard clinical guidelines for metastatic CRPC. However, the clinical decision was made on the basis of suspected neuroendocrine or undifferentiated features revealed by the lymph node biopsy and the rapidly worsening disease. In such contexts, platinum‐based chemotherapy has been documented as a potentially efficacious approach. The efficacy of this individualized treatment approach is substantiated by its alignment with the prevailing clinical context.

This report is limited by the single‐patient design, which restricts the generalizability of the findings. Furthermore, comprehensive genomic profiling was not conducted in this patient. Although molecular characterization may provide important mechanistic insights into the tumor responsiveness to platinum‐based chemotherapy, such analyses are not routinely performed in real‐world clinical practice and are even more rare in patients with rapidly progressive disease requiring immediate therapeutic intervention. In the present case, the patient′s clinical condition necessitated urgent treatment initiation, and it was not possible to obtain sufficient tumor tissue for additional molecular testing. Furthermore, at the time of treatment, access to comprehensive genomic testing was limited within our institutional framework. Importantly, this case report is primarily aimed at highlighting a clinically meaningful and durable response to carboplatin and paclitaxel in a patient with aggressive, undifferentiated CRPC under real‐world conditions, rather than establishing a definitive molecular mechanism of response. Nevertheless, the lack of any molecular profiling limits us from drawing deeper conclusions regarding the biological basis of treatment response. In addition, the long‐term efficacy and safety of prolonged carboplatin and paclitaxel therapy remain uncertain. Further accumulation of similar cases will be required to confirm the reproducibility of the study findings, as well as to refine patient selection for platinum‐based regimens.

In conclusion, the combination of carboplatin and paclitaxel represents a promising therapeutic option for patients with undifferentiated CRPC, particularly in cases where standard therapies have failed. This case contributes to the growing evidence supporting the efficacy of this regimen for managing aggressive prostate cancer phenotypes. However, further studies are necessary to fully elucidate the mechanisms underlying this therapeutic response and to identify biomarkers that can predict which patients will derive the most benefit from this treatment approach.

## Funding

No funding was received for this manuscript.

## Consent

The patient provided informed consent for the publication of this report, and the study design did not require approval by an ethics review board.

## Conflicts of Interest

The authors declare no conflicts of interest.

## Data Availability

The data that support the findings of this study are available from the corresponding author upon reasonable request.
